# Identification of candidate genes responsible for innate fear behavior in the chicken

**DOI:** 10.1093/g3journal/jkac316

**Published:** 2022-12-01

**Authors:** Takayuki Ochiai, Marina Sakaguchi, Shin-Ichi Kawakami, Akira Ishikawa

**Affiliations:** Laboratory of Animal Genetics and Breeding, Graduate School of Bioagricultural Sciences, Nagoya University, Chikusa-ku, Nagoya 464-8601, Japan; Laboratory of Animal Genetics and Breeding, Graduate School of Bioagricultural Sciences, Nagoya University, Chikusa-ku, Nagoya 464-8601, Japan; Laboratory of Animal Behavior and Physiology, Graduate School of Integrated Sciences for Life, Hiroshima University, Higashi-Hiroshima, Hiroshima 739-8528, Japan; Laboratory of Animal Genetics and Breeding, Graduate School of Bioagricultural Sciences, Nagoya University, Chikusa-ku, Nagoya 464-8601, Japan

**Keywords:** QTL, candidate gene, chicken, innate fear behavior

## Abstract

Identifying the genes responsible for quantitative traits remains a major challenge. We previously found a major QTL on chromosome 4 affecting several innate fear behavioral traits obtained by an open-field test in an F_2_ population between White Leghorn and Nagoya breeds of chickens (*Gallus gallus*). Here, an integrated approach of transcriptome, haplotype frequency, and association analyses was used to identify candidate genes for the QTL in phenotypically extreme individuals selected from the same segregating F_2_ population as that used in the initial QTL analysis. QTL mapping for the first principal component, which summarizes the variances of all affected behavioral traits in the F_2_ population, revealed the behavioral QTL located at 14–35 Mb on chromosome 4 with 333 genes. After RNA-seq analysis using two pooled RNAs from extreme F_2_ individuals, real-time qPCR analysis in the two parental breeds and their F_1_ individuals greatly reduced the number of candidate genes in the QTL interval from 333 to 16 genes. Haplotype frequency analysis in the two extreme F_2_ groups further reduced the number of candidate genes from 16 to 11. After comparing gene expression in the two extreme groups, a conditional correlation analysis of diplotypes between gene expression and phenotype of extreme individuals revealed that *NPY5R* and *LOC101749214* genes were strong candidate genes for innate fear behavior. This study illustrates how the integrated approach can identify candidate genes more rapidly than fine mapping of the initial QTL interval and provides new information for studying the genetic basis of innate fear behavior in chickens.

## Introduction

Large numbers of quantitative trait loci (QTLs) affecting traits of agricultural, medical, and biological importance have been mapped to chromosomal regions across most of the genomes of animals including livestock, poultry, model animals, and humans. However, it is difficult to identify causal genes or causal genetic variants for common QTLs with relatively small phenotypic effects, as reviewed previously (Mackay 2001; Keane et al. 2011; Albert and Kruglyak 2015; Ishikawa 2017; Visscher et al. 2017). QTLs can usually be identified either by conventional genome-wide QTL analysis based on linkage mapping in a three-generation crossbred population or by a genome-wide association study based on linkage disequilibrium in a single outbred population. Due to linkage disequilibrium, the genomic interval of each identified QTL is generally not small enough to positionally pinpoint a single candidate gene for the QTL. In recent years, various QTL mapping methods have been developed utilizing next-generation sequencing (Durbin et al. 2010; Daetwyler et al. 2014; Akbari et al. 2021), which can map QTLs more precisely than the conventional methods described above. However, most single nucleotide polymorphisms (SNPs) identified by conventional methods and next-generation sequencing are located in noncoding regions that usually contain elements that regulate gene expression, such as promoters, enhancers, and *cis*-regulatory elements. Identification of causal genes for QTLs therefore remains a major challenge. Recently, it has become increasingly clear that a single analysis is not sufficient for prioritizing candidate genes for the QTL and that different analyses need to be integrated to obtain multiple lines of evidence that consistently support one or a few candidate genes (Mackay 2001; Ishikawa 2017).

The above-described situation applies to the behavioral traits of chickens (*Gallus gallus*). Many behavioral QTLs have been reported (Buitenhuis et al. 2004; Schütz et al. 2004; Johnsson et al. 2016; Fogelholm et al. 2019; Ishikawa et al. 2020), and most of the QTLs are deposited in the Chicken QTL Database (Hu et al. 2019). Approximately 15 candidate genes for anxiety and tonic immobility behaviors were identified in QTL analyses, followed by advanced intercross lines between wild and domestic chickens (Johnsson et al. 2016; Fogelholm et al. 2019). However, no causal genes for behavioral QTLs have been reported in chickens.

We previously identified QTLs affecting innate open-field behavior on chicken chromosomes 2, 4, and 7 in an F_2_ intercross population between the G line of the White Leghorn breed (WL-G) and the native Japanese Nagoya breed (NAG; Ishikawa et al. 2020). Among the QTLs, the chromosome 4 QTL was localized in the 95% confidence interval between 14 and 35 Mb and affected seven open-field traits, suggesting that it is a major open-field locus. Interestingly, the allele derived from NAG increased open-field activity, even though NAG was more sensitive than WL-G to open-field fear (Sakaguchi and Ishikawa 2020).

In the present study, to identify candidate genes for the chromosome 4 QTL described above, we conducted an integrated approach of transcriptome analysis, haplotype frequency analysis, and two association analyses using phenotypically extreme individuals selected from the same F_2_ population as that used in the initial QTL analysis. The integrated approach eliminated the need to develop additional new crossbreeding populations for fine mapping of the initial QTL interval, which is time-consuming and labor-intensive, and successfully identified two strong candidate genes for the QTL.

## Materials and methods

### Animals

The NAG, WL-G, F_1_, and F_2_ chicks used in this study were previously produced (Ishikawa et al. 2020; Sakaguchi and Ishikawa 2020). After conducting the open-field test at 1 day of age, the body weight of the chicks was recorded, and the diencephalon was collected from each chick euthanized by decapitation and stored at −80°C. Chicks were given water from hatching to 1 day of age, but not food (Ishikawa et al. 2020; Sakaguchi and Ishikawa 2020). All chicks used in this study were handled in accordance with the guidelines of the Animal Research Committee of Nagoya University. All animal experiments were approved by the Animal Research Committee at the Graduate School of Bioagricultural Sciences, Nagoya University (authorization number AGR2019016) and conducted in accordance with the committee's guidelines. This study was also performed in compliance with the ARRIVE guidelines.

### Open-field test

The open-field test was previously performed on F_2_ chicks at 1 day after hatching (Ishikawa et al. 2020). Briefly, each chick was placed in the lower left corner of a novel arena (54 cm × 79 cm × 30 cm) that had a periphery zone within 15 cm from the edge of the arena and a center zone (39 cm × 64 cm) inside the periphery zone. The behavior of each chick was videotaped for 10 minutes, and the videotaped recordings were analyzed using SMART v3.0 software (Panlab Harvard Apparatus, California, USA) to obtain data on 14 behavioral traits. The 14 traits obtained were as follows: number of entries in the center zone, latency of the first entrance to the center zone, total time in the center zone, distance in the periphery zone, distance in the center zone, total distance, resting time, slow time, fast time, mean speed, mean speed without resting, maximum speed, parallel index, and number of excrements.

Among the 14 traits obtained, only seven traits that were affected by the QTL (Ishikawa et al. 2020) were used in this study. Of the seven traits, four traits (total distance, resting time, mean speed, and mean speed without resting) were significantly affected and the other three traits (distance in the periphery zone, slow time, and fast time) were suggestively affected. Whether raw data for the seven traits are statistically affected by environmental factors such as sex and body weight has been tested previously (Ishikawa et al. 2020). Since the previous test found that only the hatching date significantly affected all seven traits, and other environmental factors such as sex and body weight had no effect at *P* < 0.05, the raw data were adjusted for hatching date. Adjusted data for seven traits from 241 F_2_ individuals (125 males and 116 females) were used in this study.

### Principal component analysis

The adjusted seven open-field trait data were subjected to principal component analysis using a correlation matrix of JMP Pro software version 15.2.1 (SAS Institute Japan Ltd., Tokyo, Japan). The first principal component scores, the second principal component scores, and loading factors for the seven traits were calculated by JMP Pro software.

### QTL analysis

Using the first principal component scores obtained above and 881 SNP markers developed previously (Ishikawa et al. 2020), a single-QTL genome scan was performed on 241 F_2_ individuals using the Haley–Knott regression method with the function scanone of R/qtl software version 3.6.3 (Broman and Sen 2009). A logarithm of odd (LOD) score was calculated at a 1-cM step. Genome-wide 1, 5, and 10% significance threshold levels were obtained by 10,000 permutation tests of R/qtl. The 95% confidence interval of a QTL detected was estimated by a 1.8-LOD drop method. The percentage of phenotypic variance explained by the QTL and the additive and dominance effects of the QTL were calculated by the function fitqtl of R/qtl. The mode of inheritance of the QTL was estimated by the degree of dominance as previously described (Kenney-Hunt et al. 2006). A two-QTL genome scan was performed using the function scan two of R/qtl. Genome-wide 10% significance threshold levels for full and additive two-QTL models were obtained by 500 permutation tests of R/qtl.

### RNA-seq analysis

Total RNA was extracted from diencephalons of the top and bottom three F_2_ individuals of each sex using Trizol reagent (Life Technologies, Japan) and the NucleoSpin RNA kit (Takara Bio, Otsu, Japan) according to the manufacturer's instructions. The diencephalon contains the hypothalamus, which contributes to the hypothalamic–pituitary–adrenal axis that controls stress and fear responses (Matteri et al. 2000). The concentration of the total RNA obtained was measured by the Quant-iT RNA Board-Range BR Assay Kit (Thermo Fisher Scientific, Tokyo, Japan) using a Qubit Fluorometer (Thermo Fisher Scientific, Tokyo, Japan). The three RNA samples were pooled by extreme groups per sex, and the two extreme pooled RNA samples of each sex were used for RNA-seq analysis. RNA-seq analysis and subsequent sequence data analysis were outsourced to Eurofins Genomics (Tokyo, Japan). Briefly, RNA-seq analysis was performed with the next-generation sequencer Illumina Hiseq4000 using pair-end sequencing of 101-bp lengths. Adapter sequences and low-quality reads were removed using the trimmomatic software version 0.36. The cleaned reads were aligned to the chicken RefSeq GRCg6a (https://www.ncbi.nlm.nih.gov/data-hub/genome/GCF_000002315.5/) using BWA software version 0.7.17 (Li and Durbin 2009). The read counts of each transcript were compared between top and bottom groups in each sex after normalization by the Trimmed Mean of M-values (TMM) method using edgeR software version 3.16.5 (Robinson et al. 2010). The genes with log_2_FC > 0.26 (>1.2-fold) and <−0.26 (<0.83-fold) in either sex and with the same expression direction in both sexes were considered to be up- and downregulated in the bottom group, respectively.

### Quantitative real-time PCR analysis

Total RNA was extracted from diencephalons of NAG (n = 10), WL-G (n = 10), F_1_ (n = 10) chickens, and top F_2_ (n = 20) and bottom F_2_ (n = 19) chickens ranked by the first principal component score. cDNA was synthesized from 1.0 µg of total RNA using the PrimerScript RT reagent Kit with gDNA Eraser (Takara Bio, Otsu, Japan) according to the manufacturer's instructions. Quantitative real-time PCR analysis was conducted by an Applied Biosystems StepOnePlus Real-Time PCR system (Thermo Fisher Scientific, Tokyo, Japan) with TB Green Premix Ex Taq II (Tli RNaseH Plus) (Takara Bio, Otsu, Japan). The thermal conditions of real-time PCR were initial denaturation at 95°C for 30 seconds, 40 cycles of denaturation at 95°C for 5 seconds, annealing and extension at 60°C for 30 seconds, and additional extension for 60 seconds. To find appropriate internal control genes among the four genes *Pol II*, *TBP*, *ACTB*, and *GAPDH*, the amplification efficiency of the target and control genes was simultaneously calculated using a quantitative relative standard curve with four concentrations of serial dilutions (10, 2, 0.4, and 0.08 ng/µl). Pairs of target and control genes that showed similar amplification efficiency were analyzed using the 2^−ΔΔCt^ method. Primer sequences are listed in Supplementary Table 1. All samples were analyzed in triplicate.

Before comparing the obtained gene expression levels among NAG, WL-G and F_1_ groups, the effects of group, sex, and their interaction on expression levels were tested using two-way analysis of variance (ANOVA) in JMP Pro software. Expression levels were adjusted only for sex, which was significant at *P* < 0.05 (Supplementary Table 2), using a linear model in JMP Pro software. Using adjusted expression levels for sex, the NAG, WL-G, and F_1_ groups were compared by one-way ANOVA followed by Tukey's honestly significant difference post hoc test in JMP Pro software. Similarly, the statistical significance of the effects of group, sex, and their interaction on expression levels were tested before comparison of expression levels between the top and bottom F_2_ groups. After adjusting the expression data for effects significant at *P* < 0.05 (Supplementary Table 3), adjusted expression levels between the two groups were compared by Student's *t*-test in JMP Pro software.

### Haplotype frequency analysis

Diplotypes of the top and bottom F_2_ individuals were determined on the basis of genotypes of 16 SNP marker loci (see Table 1) within the 95% confidence interval of the above-mapped QTL. The marker loci were previously developed and simultaneously genotyped by RAD-seq analysis (Ishikawa et al. 2020). Haplotype frequencies were determined on the basis of diplotypes and compared between the two groups of top and bottom F_2_ individuals by Pearson's chi-square test. F_2_ individuals with recombinant haplotypes were excluded from the test.

**Table 1.** A list of 35 genes that tend to be differentially expressed in the 95% confidence interval of the chromosome 4 QTL detected by RNA-seq analysis.

Gene	Position (bp)	Log_2_FC^a^	
		Male	Female
*ZBTB33*	16,473,770–16,484,173	−0.14	−0.40
***NDUFA1***	16,493,404–16,494,171	0.30	0.27
*NKRF*	16,532,474–16,542,429	0.53	0.07
***SLC25A43***	16,562,212–16,583,458	−0.52	−0.23
***PASD1***	17,394,750–17,501,799	−0.09	−0.27
*GPR50*	17,602,861–17,634,184	−2.03	−0.76
*MAMLD1*	17,785,536–17,877,708	−0.10	−0.34
***TMEM185A***	18,045,407–18,057,071	0.18	0.38
*LOC112532367*	18,108,763–18,120,433	0.05	0.32
***TLR2A***	20,006,893–20,028,956	0.57	0.40
*SFRP2*	20,063,343–20,067,714	−0.30	−0.75
*LRAT*	20,396,173–20,399,224	0.81	0.33
*NPY2R*	20,537,583–20,547,575	0.27	0.19
***FAM198B***	21,766,896–21,787,710	−1.24	−0.08
*NAF1*	23,312,484–23,342,398	0.18	0.48
***NPY5R***	23,386,250–23,395,197	−0.30	−0.54
*APELA*	23,781,390–23,786,479	0.60	0.36
***TLL1***	24,058,915–24,221,046	0.03	0.34
*PALLD*	25,014,616–25,202,319	−0.45	−0.21
***AADAT***	25,220,146–25,235,204	0.15	0.42
*MFAP3L*	25,261,123–25,283,188	−0.28	−0.02
***NAA15***	29,258,294–29,298,615	−0.08	−0.37
*LOC422442*	29,323,717–29,327,017	−0.30	−0.39
*LOC112532278*	29,766,125–29,771,905	−0.42	−0.11
***LOC101749214***	31,375,703–31,405,998	0.13	0.28
*SLC10A7*	31,386,080–31,566,794	−0.06	−0.45
*LOC107051782*	32,317,753–32,365,112	−0.40	−0.73
*MAB21L2*	33,007,846–33,009,573	0.36	0.40
*SLC20A2*	34,163,904–34,231,930	−0.27	−0.15
***PLK4***	34,340,512–34,358,347	−0.31	−0.16
***MFSD8***	34,356,519–34,369,982	−0.09	−0.32
*JADE1*	34,537,748–34,598,398	0.33	0.13
***BTC***	34,616,757–34,622,370	0.42	0.87
***MMRN1***	35,448,636–35,506,195	−0.05	−0.99
***CCSER1***	35,498,952–36,242,028	−0.03	−0.50

The physical map positions are based on the chicken RefSeq GRCg6a. SNP marker loci located on the 16 genes in bold were used for haplotype frequency analysis of top and bottom F_2_ individuals.

Fold change.

### Correlation analysis

To eliminate the influence of diplotype, a conditional correlation analysis between gene expression levels and the first principal component scores of top and bottom 39 F_2_ individuals combined was performed using a linear model conditional on diplotype in JMP Pro software.

### Sequence analysis

In two pooled F_2_ RNAs used for RNA-seq analysis, a synonymous SNP was found in the coding region of *NPY5R*, a candidate gene for the QTL, as described below. The SNP genotypes of NAG and WL-G breeds were determined by direct sequence analysis of PCR products amplified from genomic DNA and cDNA. Genomic DNA was extracted from the blood of one NAG male and four WL-G females used as direct grandparents of the F_2_ QTL mapping population (Ishikawa et al. 2020) and 10 F_1_ individuals (n = 5 of each sex) used for RT-qPCR analysis, using a DNeasy Blood and Tissue kit (Qiagen, Tokyo, Japan) according to the manufacturer's protocol. The 205-bp SNP region of genomic DNA and cDNA was amplified on an Applied Biosystems Veriti Thermal Cycler (Thermo Fisher Scientific, Tokyo, Japan) using a pair of primers (Supplementary Table 1) and Quick Taq HS DyeMix (Toyobo, Osaka). PCR conditions were 94°C for 2 minutes, followed by 40 cycles of 94°C for 30 seconds, 55°C for 30 seconds, and 68°C for 1 minute, and 68°C for 20 seconds. The PCR products were purified with a Wizard SV Gel and PCR Clean-Up System (Promega, Tokyo, Japan). Sequence analysis of the PCR products purified was outsourced to Eurofins Genomics (Tokyo, Japan). Both strands of the PCR products were determined on an Applied Biosystems 3730xl DNA Analyzer (Thermo Fisher Scientific, Tokyo, Japan) using a BigDy Terminator v3.1 Cycle Sequencing kit (Thermo Fisher Scientific, Tokyo, Japan).

### Allelic imbalance analysis

Using the genomic DNA and cDNA sequence data of 10 F_1_ individuals obtained above, the relative peak heights of sequence waveforms were manually measured according to the method described by Ge et al. (2005) to examine the imbalance between the two SNP alleles of *NPY5R* expression. For both genomic DNA and cDNA, the ratio of relative peak heights of mutant to wild-type SNP alleles was calculated. Mean height ratios among the four groups by DNA and sex combination were compared with Kruskal–Wallis test followed by Steel–Dwass post hoc test in JMP Pro software because the group variances were not equal at *P* < 0.0001 (Welch test). In the absence of imbalance between the two alleles, the peak height ratio was expected to be 1. The mean peak height ratio of each of the genomic DNA and cDNA were compared with the expected value of 1 by Wilcoxon signed-rank test in JMP Pro software.

## Results

**Fig. 1.**
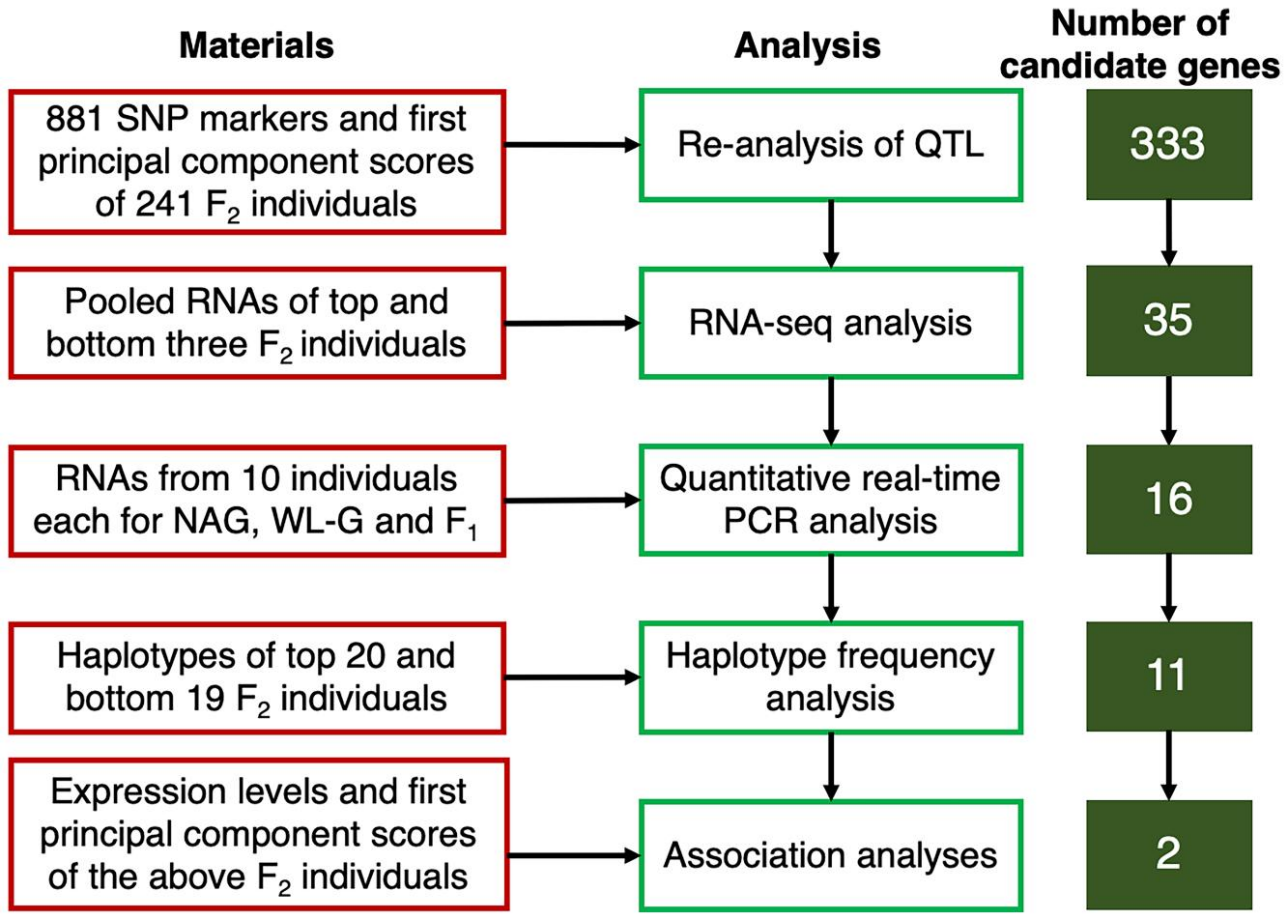
Overview of an integrated approach for identification of candidate genes for the open-field QTL on chicken chromosome 4 using a segregating F_2_ population between WL-G and NAG chicken breeds.

Using 241 F_2_ individuals between WL-G and NAG, we integrated five analyses and performed them sequentially to identify strong candidate genes for the open-field QTL on chicken chromosome 4. The workflow diagram of the integrated approach is shown in Fig. 1.

### Re-analysis of the QTL map location

**Fig. 2.**
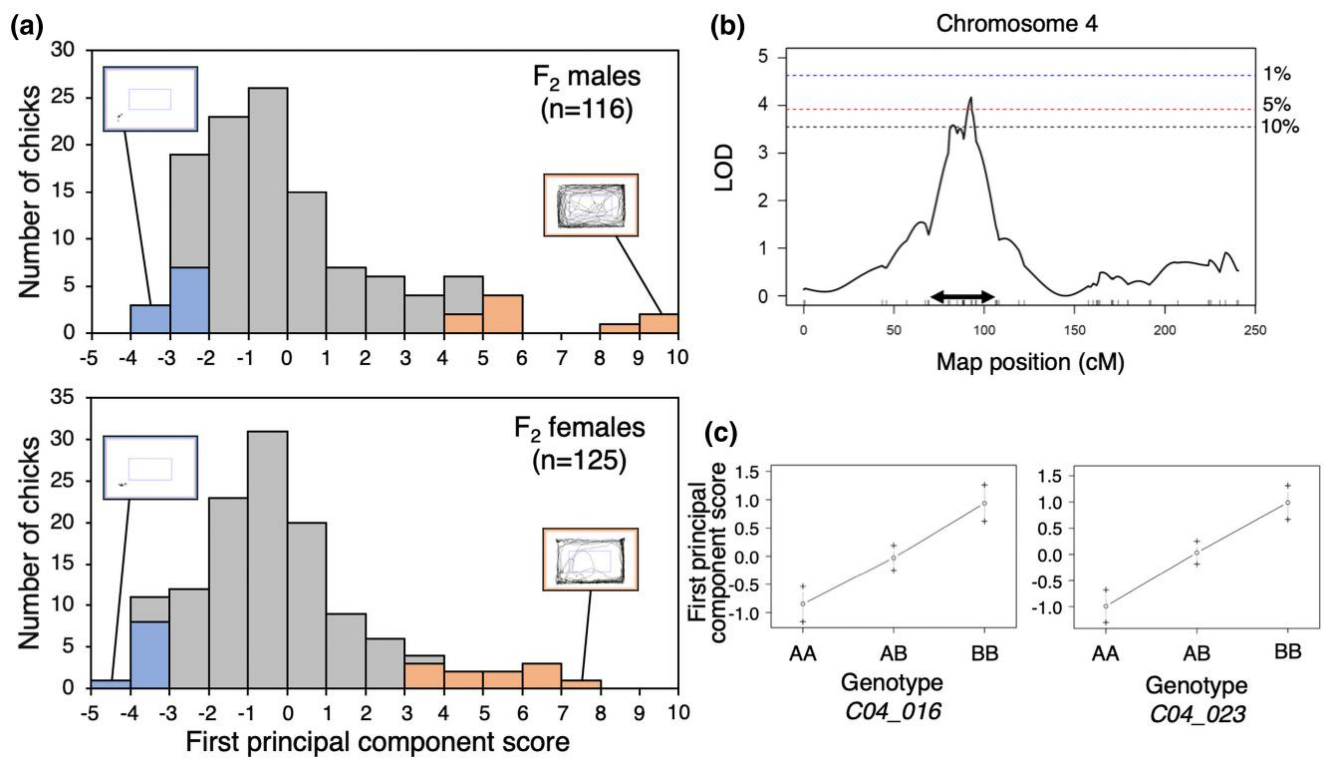
Results of QTL analysis for the first principal component scores for seven open-field traits in an F_2_ population between WL-G and NAG breeds: a) histograms showing the distribution of the first principal component scores by sex. The orange and blue bars indicate top and bottom individuals, respectively, that were used for gene expression analysis. Insets show movement trajectories of typical top and bottom individuals in the open-field arena. b) LOD curve plot on chromosome 4. The blue, red, and black dashed horizontal lines indicate genome-wide 1, 5, and 10% significance threshold levels, respectively, obtained by 10,000 permutation tests. The double-headed arrow shows the 95% confidence interval of the LOD peak. c) Effect plots (mean ± SEM) for the *C04_016* and *C04_023* SNP markers nearest the LOD peaks that exceeded 10 and 5% levels, respectively. The letters A and B represent alleles derived from WL-G and NAG breeds, respectively.

To confirm the exact map location of the open-field QTL on chromosome 4, we performed principal component analysis, followed by QTL analysis, using only seven open-field traits affected by the QTL. Principal component analysis revealed that the first and second axes of principal components explained 90.2 and 6.2% of the total open-field variance, respectively (Supplementary Fig. 1). For the first principal component, resting time had a negative factor loading and the other six traits had positive factor loadings, indicating that F_2_ chicks with higher positive first principal component scores were more active in a novel open-field arena than those with lower negative first principal component scores. As shown in their movement trajectories shown in Fig. 2a, the top-ranking F_2_ individuals in the distribution of the first principal component scores were very active in the open-field arena and the bottom-ranking F_2_ individuals were inactive for both sexes. Only the first principal component scores that explained most of the total variance were used as a quantitative trait for genome-wide QTL analysis described below.

Using the first principal component scores and 881 SNP markers, simple interval mapping was performed on the 241 F_2_ individuals by the function scanone of R/qtl software. The LOD scores for genome-wide 1, 5, and 10% significance threshold levels were estimated to be 4.6, 3.9, and 3.6, respectively. A QTL with a peak LOD score of 4.2 exceeded the genome-wide 5% threshold level, and it was detected at 93 cM on chromosome 4 (Fig. 2b), at which the nearest SNP marker *C04_023* (29.5 Mb) was located. The 95% confidence interval of the QTL was estimated to be 14–35 Mb. This QTL explained 7.7% of the phenotypic variance. The additive and dominance effects (mean ± SEM) in standard deviation units were 1.00 ± 0.23 and 0.02 ± 0.31, respectively. The degree of dominance (ratio of the dominance effect to the additive effect) was calculated to be 0.02, clearly showing an additive mode of inheritance of the QTL. The NAG-derived allele at the *C04_023* marker locus increased the first principal component score (Fig. 2c).

An additional suggestive LOD peak (LOD = 3.6) exceeding the genome-wide 10% level was located at 83 cM near the *C04_016* marker locus (24.7 Mb) on chromosome 4 (Fig. 2b). The NAG-derived allele at the *C04_016* locus increased the first principal component score (Fig. 2c). To confirm the presence of the suggestive QTL, a two-QTL genome scan was performed by the function scan two of R/qtl. The genome-wide 10% significance threshold levels for the full and additive models of the two-QTL scan were estimated to be 8.7 and 4.3, respectively. No statistical evidence showing the presence of the additional suggestive QTL (LOD = 2.3 and 0.7) was obtained at the 10% threshold levels.

### RNA-seq analysis

Based on the chicken RefSeq GRCg6a, 333 genes were present in the 95% confidence interval of the open-field QTL on chromosome 4. To find genes that tended to be differentially expressed among the 333 genes, RNA-seq analysis was performed using pooled diencephalic RNAs extracted from the top and bottom three F_2_ individuals ranked by the first principal component score in each sex. In total, 70,569,862 reads were obtained and approximately 78% of the reads were mapped to chicken RefSeq GCRg6a. RNA-seq analysis revealed that 35 genes tended to be differentially expressed in the 95% confidence interval (Table 1). Among the 35 genes, 15 tended to be upregulated and 20 tended to be downregulated in the bottom group. The *LOC112532278* gene, which tended to be differentially expressed, was excluded from the next prioritization analysis because it completely overlapped on the physical map with the non-differentially expressed gene *RNF150*, making it difficult to design primers that accurately quantify the expression of *LOC112532278* and *RNF150* separately.

### Quantitative real-time PCR analysis

To confirm the expression of the 34 genes (excluding *LOC112532278*), quantitative real-time PCR analysis was performed in NAG and WL-G and their F_1_ chicks. Two-way ANOVA revealed that 13 genes had significant sex effects on the expression levels, but no genes had significant breed-by-sex interaction effects at nominal *P* < 0.05 (Supplementary Table 2). After adjusting the expression levels of 13 genes for sex, it was found that 16 genes showed significant differential expression levels in NAG, WL-G, and F_1_ chicks (Table 2). One-way ANOVA revealed that the expression of three genes (*TLL1*, *AADAT*, and *LOC101749214*) was significantly upregulated in WL-G compared with that in NAG at *P* < 0.05 (Tukey's honestly significant difference test). Conversely, the expression of 10 genes (*NDUFA1*, *SLC25A43*, *PASD1*, *TMEM185A*, *TLR2A*, *FAM198B*, *NPY5R*, *MFSD8*, *MMRN1*, and *CCSER1*) was significantly downregulated in WL-G. For these 13 up- and downregulated genes, F_1_ expression was between the two parental breeds. However, *NAA15*, *PLK4*, and *BTC* expression levels were not significantly different between the two parental breeds. The F_1_ expression for *PLK4* and *BTC* was significantly different from the expression in either parental breed. Uniquely, the *NAA15* expression in F_1_ was significantly lower than that in both parental breeds. No significant differences in expression were found for the remaining 18 genes (Table 2).

**Table 2.** Diencephalic expression levels of 34 genes quantified by quantitative real-time PCR analysis using NAG, WL-G, and their F_1_ chicks.

Gene	NAG	F_1_	WL-G	*P* value
*ZBTB33*	1 ± 0.05	0.99 ± 0.05	1.11 ± 0.05	0.24
*NDUFA1*	1 ± 0.07ª	0.47 ± 0.07^b^	0.11 ± 0.07^c^	1.8 × 10^−09^
*NKRF*	1 ± 0.06	0.92 ± 0.08	0.78 ± 0.07	0.091
*SLC25A43*	1 ± 0.05^a^	0.73 ± 0.05^b^	0.56 ± 0.05^b^	4.5 × 10^−06^
*PASD1*	1 ± 0.06ª	0.93 ± 0.06^ab^	0.79 ± 0.06^b^	0.042
*GPR50*	1 ± 0.05	0.98 ± 0.05	1.01 ± 0.05	0.37
*MAMLD1*	1 ± 0.06	0.97 ± 0.06	1.08 ± 0.06	0.38
*LOC112532367*	1 ± 0.14	0.95 ± 0.14	0.76 ± 0.14	0.44
*TMEM185A*	1 ± 0.07ª	0.73 ± 0.07^b^	0.67 ± 0.07^b^	0.0042
*TLR2A*	1 ± 0.06ª	0.84 ± 0.06^ab^	0.77 ± 0.06^b^	0.024
*SFRP2*	1 ± 0.12	0.88 ± 0.12	0.73 ± 0.12	0.29
*LRAT*	1 ± 0.04	1.01 ± 0.05	0.93 ± 0.06	0.49
*NPY2R*	1 ± 0.06	0.96 ± 0.06	0.81 ± 0.06	0.077
*FAM198B*	1 ± 0.05ª	0.63 ± 0.05^b^	0.49 ± 0.05^b^	1.3 × 10^−07^
*NAF1*	1 ± 0.06	1.00 ± 0.06	1.03 ± 0.06	0.93
*NPY5R*	1 ± 0.04ª	0.79 ± 0.04^b^	0.79 ± 0.04^b^	3.9 × 10^−04^
*APELA*	1 ± 0.05	1.04 ± 0.05	0.95 ± 0.05	0.45
*TLL1*	1 ± 0.04^a^	1.02 ± 0.04^ab^	1.16 ± 0.04^b^	0.021
*PALLD*	1 ± 0.05	0.89 ± 0.05	0.87 ± 0.05	0.19
*AADAT*	1 ± 0.06^a^	1.22 ± 0.06^b^	1.26 ± 0.06^b^	0.015
*MFAP3L*	1 ± 0.07	0.91 ± 0.07	0.96 ± 0.07	0.62
*NAA15*	1 ± 0.04ª	0.78 ± 0.04^b^	0.92 ± 0.04^a^	6.8 × 10^−04^
*LOC422442*	1 ± 0.08	0.94 ± 0.08	0.77 ± 0.08	0.11
*LOC101749214*	1 ± 0.04^a^	1.11 ± 0.40^ab^	2.43 ± 0.40^b^	0.033
*SLC10A7*	1 ± 0.05	0.84 ± 0.05	0.92 ± 0.05	0.068
*LOC107051782*	1 ± 0.08	0.98 ± 0.08	0.80 ± 0.08	0.13
*MAB21L2*	1 ± 0.08	1.13 ± 0.08	0.87 ± 0.08	0.073
*SLC20A2*	1 ± 0.08	1.17 ± 0.08	0.96 ± 0.08	0.15
*PLK4*	1 ± 0.06ª	0.76 ± 0.06^ab^	0.79 ± 0.06^b^	0.016
*MFSD8*	1 ± 0.07ª	0.77 ± 0.07^a^	0.50 ± 0.07^b^	2.1 × 10^−04^
*JADE1*	1 ± 0.08	0.99 ± 0.08	0.78 ± 0.08	0.10
*BTC*	1 ± 0.10^a^	1.49 ± 0.10^b^	1.18 ± 0.10^ab^	0.0037
*MMRN1*	1 ± 0.16^a^	2.09 ± 0.16^b^	3.00 ± 0.16^c^	8.1 × 10^−09^
*CCSER1*	1 ± 0.03ª	0.85 ± 0.03^b^	0.84 ± 0.03^b^	0.0014

Data (n = 10/breed) are presented as mean ± SEM. The data were adjusted for sex, when the effect of sex on the expression data was significant at nominal *P* < 0.05 (Supplementary Table 2). The *P* values were obtained by one-way ANOVA.

Means with different letters are significantly different between the two groups at *P* < 0.05 by Tukey's honestly significant difference test.

### Haplotype frequency analysis

If causal gene(s) for the open-field QTL were involved in the 95% confidence interval, the frequencies of haplotypes derived from NAG and WL-G for the confidence interval should differ significantly between the top 20 and bottom 19 F_2_ chicks ranked by the first principal component score. Haplotype frequency analysis was performed for the 16 loci confirmed by real-time PCR analysis using the two extreme F_2_ groups after the removal of individuals with haplotype recombination. At 11 loci (*FAM198B*, *NPY5R*, *TLL1*, *AADAT*, *NAA15*, *LOC101749214*, *PLK4*, *MFSD8*, *BTC*, *MMRN1*, and *CCSER1*), the haplotype frequencies calculated from three diplotypes, NAG/NAG, NAG/WL, and WL/WL, were significantly different between the two extreme groups (Table 3). As expected, the frequency of the NAG haplotype was significantly higher in the top group than in the bottom group. At the other five loci, no significant differences in haplotype frequency were observed between the two groups (Table 3).

**Table 3.** Haplotype frequencies calculated from three diplotypes at 16 loci between the top and bottom F_2_ groups.

Gene	Haplotype	Top	Bottom	Chi-square value	*P* value
*NDUFA1*	NAG	0.62 (21)	0.40 (12)	3.02	0.082
	WL-G	0.38 (13)	0.60 (18)		
*SLC25A43*	NAG	0.62 (21)	0.40 (12)	3.02	0.082
	WL-G	0.38 (13)	0.60 (18)		
*PASD1*	NAG	0.62 (21)	0.40 (12)	3.02	0.082
	WL-G	0.38 (13)	0.60 (18)		
*TLR2A*	NAG	0.65 (22)	0.42 (15)	3.73	0.054
	WL-G	0.35 (12)	0.58 (21)		
*TMEM185A*	NAG	0.65 (22)	0.42 (15)	3.73	0.054
	WL-G	0.35 (12)	0.58 (21)		
*FAM198B*	NAG	0.72 (26)	0.42 (15)	6.85	0.0088
	WL-G	0.28 (10)	0.58 (21)		
*NPY5R*	NAG	0.72 (26)	0.42 (15)	6.85	0.0088
	WL-G	0.28 (10)	0.58 (21)		
*TLL1*	NAG	0.72 (26)	0.42 (15)	6.85	0.0088
	WL-G	0.28 (10)	0.58 (21)		
*AADAT*	NAG	0.72 (26)	0.44 (15)	5.69	0.017
	WL-G	0.28 (10)	0.56 (19)		
*NAA15*	NAG	0.76 (26)	0.44 (14)	7.39	0.0066
	WL-G	0.24 (8)	0.56 (18)		
*LOC101749214*	NAG	0.74 (25)	0.44 (16)	6.10	0.014
	WL-G	0.26 (9)	0.56 (20)		
*PLK4*	NAG	0.74 (25)	0.44 (16)	6.10	0.014
	WL-G	0.26 (9)	0.56 (20)		
*MFSD8*	NAG	0.74 (25)	0.44 (16)	6.10	0.014
	WL-G	0.26 (9)	0.56 (20)		
*BTC*	NAG	0.74 (25)	0.44 (16)	6.10	0.014
	WL-G	0.26 (9)	0.56 (20)		
*MMRN1*	NAG	0.68 (27)	0.44 (16)	4.10	0.043
	WL-G	0.33 (13)	0.56 (20)		
*CCSER1*	NAG	0.68 (27)	0.44 (16)	4.10	0.043
	WL-G	0.33 (13)	0.56 (20)		

The number of haplotypes is shown in parentheses. Individuals with haplotype recombination were excluded from the analysis. The *P* values were obtained by Pearson's chi-square test (*df* = 1).

### Association analyses

**Fig. 3.**
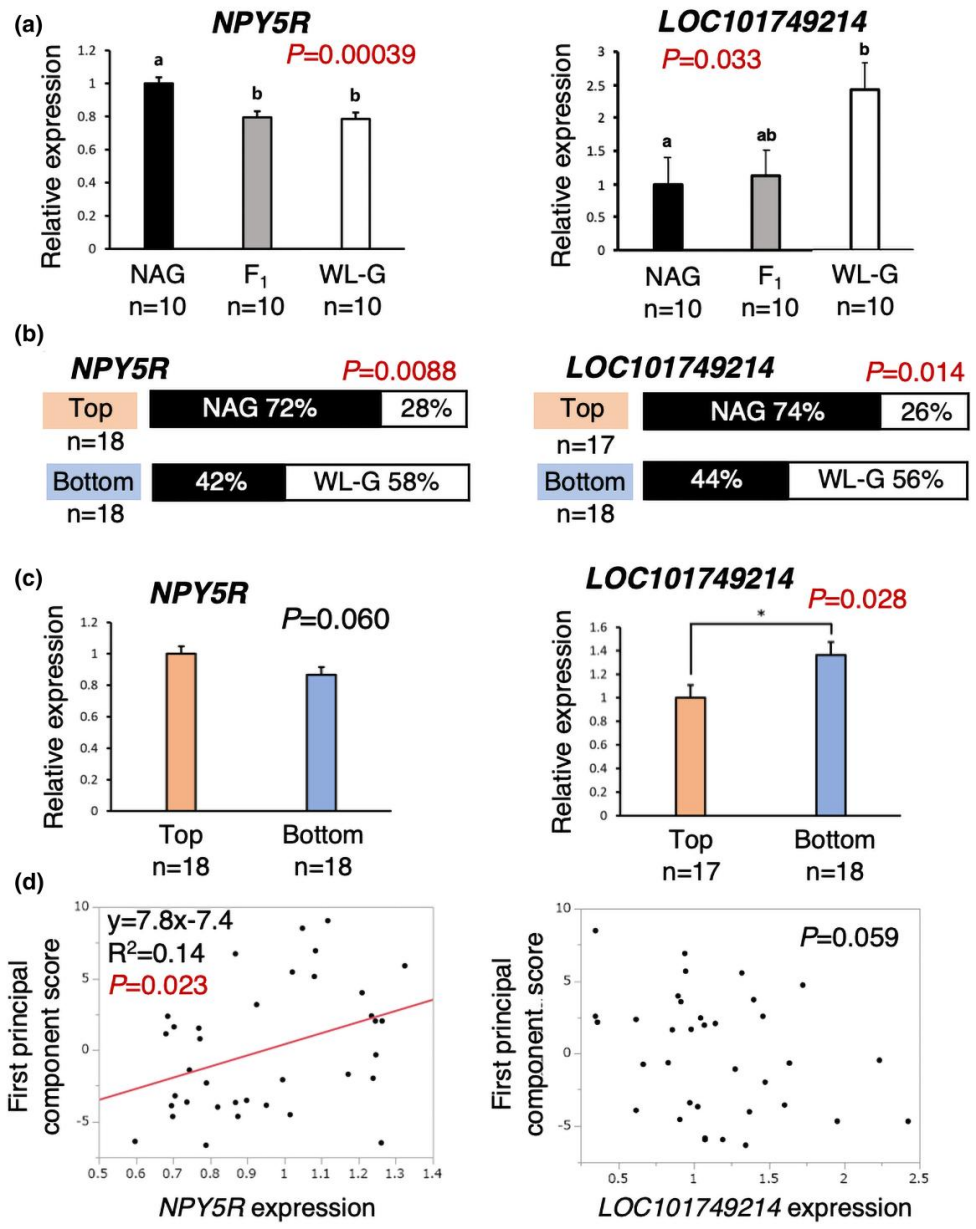
Results of four analyses for *NPY5R* and *LOC101749214* genes: a) quantitative real-time PCR analysis in NAG, WL-G, and their F_1_ hybrids. Data are presented as ± SEM. *P* values were obtained by one-way ANOVA. The means with different letters (a and b) were significantly different between the two groups at *P* < 0.05 (Tukey's honestly significant difference test). b) Analysis of haplotype frequency between the top and bottom F_2_ groups. *P* values were obtained by the chi-square test. c) Quantitative real-time PCR analysis in the top and bottom F_2_ groups. *P* values were obtained by Student's *t*-test. d) A conditional correlation analysis of diplotypes between gene expression levels and the first principal component scores for top and bottom F_2_ individuals combined.

For the 11 genes that passed the above haplotype analysis, two association analyses were conducted using the top 20 and bottom 19 F_2_ individuals. One analysis was carried out to compare expression levels in the diencephalon between the two extreme F_2_ groups, while the other analysis was carried out to examine the conditional correlation of diplotypes between the expression levels and first principal component scores of the 39 extreme F_2_ individuals combined. In the former expression analysis, only *LOC101749214* out of the 11 genes was significantly upregulated in the bottom group compared with that in the top group at *P* < 0.05 (Student's *t*-test; Table 4; Fig. 3c). *NPY5R* expression was marginally significant, with a trend toward lower expression in the bottom group than in the top group. For the other nine genes, no significant differences in expression were found between the two extreme groups (Table 4). In the latter conditional correlation analysis of diplotypes, among the 11 genes, only *NPY5R* showed a significant positive correlation between the expression levels and the first principal component scores (Fig. 3d). *LOC101749214* expression showed a marginal negative correlation with the first principal component scores. For the other nine genes, no significant correlations were detected between gene expression and first principal component scores (Supplementary Fig. 2).

**Table 4.** Diencephalic expression levels of 11 genes in the top and bottom F_2_ groups.

Gene	Top	Bottom	*P* value
*FAM198B*	1 ± 0.06 (18)	0.88 ± 0.06 (18)	0.18
*NPY5R*	1 ± 0.05 (18)	0.87 ± 0.05 (18)	0.060
*TLL1*	1 ± 0.06 (18)	0.96 ± 0.06 (18)	0.69
*AADAT*	1 ± 0.08 (18)	1.10 ± 0.08 (17)	0.39
*NAA15*	1 ± 0.09 (17)	0.95 ± 0.09 (16)	0.71
*LOC101749214*	1 ± 0.11 (17)	1.37 ± 0.11 (18)	0.028
*PLK4*	1 ± 0.10 (17)	0.94 ± 0.09 (18)	0.67
*MFSD8*	1 ± 0.07 (17)	1.00 ± 0.07 (18)	0.98
*BTC*	1 ± 0.09 (17)	1.06 ± 0.09 (18)	0.65
*MMRN1*	1 ± 0.11 (20)	1.18 ± 0.12 (18)	0.28
*CCSER1*	1 ± 0.04 (20)	0.99 ± 0.04 (18)	0.81

Data are presented as mean ± SEM. The number of individuals is shown in parentheses. The data were adjusted for group, sex, and/or sex-by-group interaction when their effects on expression were significant at nominal *P* < 0.05 (Supplementary Table 3). The *P* values were obtained by Student's *t*-test.

By the integrated analyses, the first 333 genes in the 95% confidence interval of the open-field QTL were successfully narrowed down to two genes of *NPY5R* and *LOC101749214*. The results of a series of analyses for the two genes are summarized in Fig. 3 and are overviewed in Fig. 1.

### SNP analysis of candidate gene coding regions

**Fig. 4.**
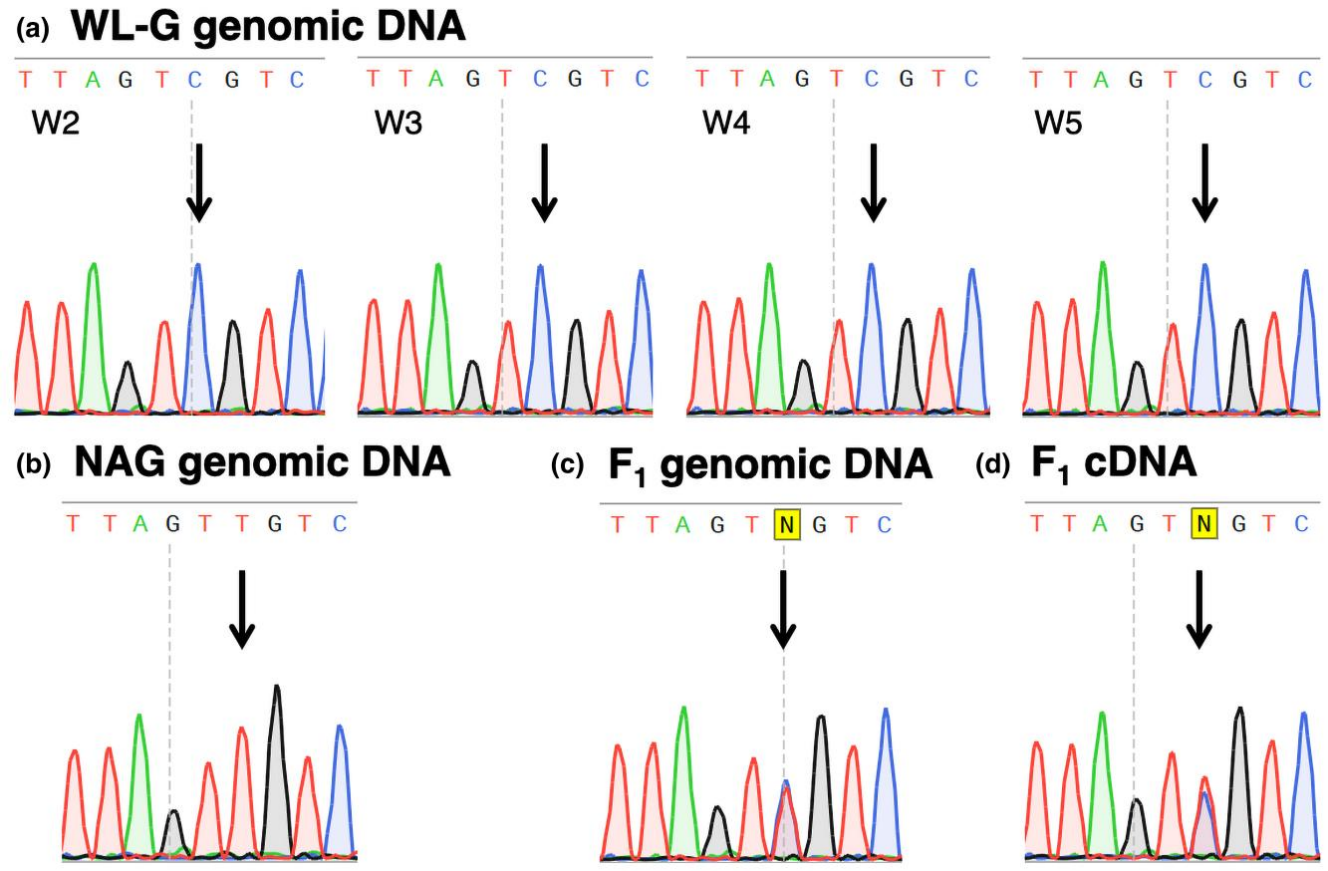
Sequence results for the SNP *rs316511723* region of the *NPY5R* gene: a) genomic DNA of four WL-G females (W#) with a clear homozygous genotype (C/C). b) Genomic DNA of a NAG Male chicken with a clear homozygous genotype (T/T). c) Genomic DNA of an F_1_ chicken obtained from a cross between the W3 WL-G female and the NAG male in (b). The F_1_ is a clear heterozygote (C/G). d) cDNA of the same F_1_ chicken. For all F_1_ chickens obtained from four WL-G females and the NAG male in (b) (Supplementary Fig. 3). Arrows show the nucleotide of the SNP.

Examination of the RNA-seq data in pooled F_2_ samples revealed no SNPs in the coding region of *LOC101749214*. However, there was only one synonymous SNP (T < C) at g: 23,392,923 bp on the *NPY5R* coding region. The top samples had the same codon GTT as the chicken RefSeq GRCg6a, encoding Valine. The bottom samples had the codon GTC encoding the same amino acid. Sequence analysis of genomic DNA from one NAG male and four WL-G females used as direct grandparents for the F_2_ population revealed that NAG was clearly homozygous for the reference allele T and WL-G for the mutant allele C (Fig. 4, a and b). This SNP was consistent with the previously reported SNP *rs316511723*.

### Allelic imbalance analysis

Sequence analysis of the *rs316511723* region of *NPY5R* was performed using genomic DNA and cDNA from 10 F_1_ individuals used for the real-time PCR analysis described earlier (Fig. 3a). Genomic DNA sequence analysis showed that all F_1_ individuals were clearly heterozygous for both C and T alleles, and these two alleles had nearly the same peak height (Fig. 4c and Supplementary Fig. 3a). On the other hand, cDNA sequence analysis showed that all 10 F_1_ individuals were heterozygous for both alleles, but the peak height of the T allele appeared to be slightly higher than that of the C allele (Fig. 4d and Supplementary Fig. 3b). Kruskal–Wallis test showed that the mean ratios of relative peak heights of the C allele to the T allele among the four groups by DNA and sex combination were significant at *P* < 0.001 (Table 5). In both sexes, the mean peak height ratio of cDNA was significantly lower than that of genomic DNA at *P* < 0.05 (Steel–Dwass test). The height ratio of both genomic DNA and cDNA did not differ significantly between the sexes (Table 5). However, there was no significant difference from the expected height ratio of 1, assuming an allelic ratio of 1:1, in any of the four groups (Wilcoxon signed-rank test).

**Table 5.** Relative peak height ratios of the C and T alleles of the *NPY5R* SNP.

Sex	Genomic DNA	cDNA
Male	1.1 ± 0.02^a^	0.8 ± 0.01^b^
Female	1.1 ± 0.02^a^	0.7 ± 0.04^b^

Data (*n* = 5/DNA/sex) are presented as mean ± SEM. The mean ratios among the four groups by DNA and sex combination were significant at *P* < 0.001 (Kruskal–Wallis test).

Means with different letters are significantly different between two of the four groups at *P* < 0.05 by Steel–Dwass test.

## Discussion

In the present study, the location of the open-field QTL on chicken chromosome 4 was re-analyzed using the first principal component scores that summarized the phenotypic variances of the seven open-field traits affected by the QTL, confirming the existence of a significant QTL that was previously mapped for individual open-field traits (Ishikawa et al. 2020). The map location and the 95% confidence interval length of the QTL were the same in the present study and the previous study. Furthermore, the NAG-derived QTL allele was confirmed to unexpectedly reduce open-field fear, implying that this QTL is not a locus for the timid temperament seen in the NAG breed.

The two-QTL genome scan did not provide statistical evidence of an additional suggestive QTL that has the same sign of the phenotypic effect as the significant QTL described earlier. However, the possibility of the existence of an additional QTL cannot be ruled out as the two-QTL scan is known to be effective in separating two linked QTLs with opposing phenotypic effects (Broman and Sen 2009). The possibility might be supported by the detection of two candidate genes: *NPY5R* and *LOC101749214*.

Using individual RNA samples for RNA-seq analysis may yield more robust results than the results from pooled RNA samples used in this study. However, pooling RNA samples would reduce differences between individual RNA samples and would facilitate obtaining gene expression levels between the two extreme groups. To minimize as much as possible the loss of power to identify differences in gene expression, we used lower fold changes (>1.2-fold and <0.83-fold) as thresholds. In addition, we performed real-time PCR experiments using different populations (NAG, WL, and F_1_). In fact, RNA-seq studies using pooled hypothalamic RNA samples have been performed to discover differences in gene expression (e.g. Sharma et al. 2018; Chen et al. 2020; Adlanmerini et al. 2021; Mohr et al. 2021).

In the present study, one synonymous SNP (T < C) was found in the coding region of *NPY5R*. Allelic imbalance analysis did not provide clear evidence of differential expression of *NPY5R* between the T and C alleles. Even if allelic imbalance did exist, no biological effect would be observed because the SNP is synonymous with no amino acid change.

Although the biological function of *LOC101749214* has not been reported, this gene is believed to overlap in the antisense direction with the ion transporter *SLC10A7* at its 3′ end. *SLC10A7* expression was marginally different among NAG, WL-G, and F_1_ chickens. On the other hand, *NPY5R* effects on fear and anxiety behaviors have been reported in rats. For example, *NPY5R* in the amygdala had an anxiolytic effect on social interaction behavior (Sajdyk et al. 2002). The infusion of the specific Y5 agonist [cPP]hPP into the paraventricular nucleus of the hypothalamus mediated a reduction in anxiety-related behavior in a light/dark box test (Martinetz et al. 2019). Rats that received an intracerebroventricular injection of the specific Y5 agonist [cPP(1–7), NPY(19–23), Ala(31), Aib(32), and Gln(34)]hPP exhibited anxiolytic-like behavior in an open-field test (Sørensen et al. 2004). These results of previous studies suggest that NPY signaling via *NPY5R* acts on regulating anxiety, and the results also indicate the difficulty in identifying the main brain regions involved in the control of anxiety-related behaviors. This difficulty may be true for chickens, since *NPY5R* has been reported to be widely expressed in various regions of the brain (He et al. 2016).

Transcriptional analysis in the hypothalamus of newly hatched chicks during fasting and delayed feeding suggested that increased *NPY5R* mRNA may be consistent with increased appetite in fasted chicks (Higgins et al. 2010). In chicks at 2 days of age, an intracerebroventricular injection of procine [Leu^31^, Pro^34^]-NPY, which has a high affinity to chicken *NPY2R* and *NPY5R*, significantly increased food intake in neonatal chicks (Ando et al. 2001), suggesting that *NPY2R* and *NPY5R* may regulate feeding behavior in chickens. In the present study, *NPY5R* expression in F_2_ chicks was positively correlated with the first principal component scores, and although F_2_ chicks were not fed from hatching until 1 day of age when the open-field test is conducted, there was no significant difference in body weights at 1 day of age, suggesting that F_2_ chicks with higher *NPY5R* expression may be more active foragers in the open-field arena.

In strategies to identify candidate genes, consideration is often given to preferential expression in tissues related to a trait of interest. However, this is not always true. In cows, *DGAT1*, a causal gene for milk yield and composition QTLs, is not preferentially expressed in tissues associated with milk production (Ron and Weller 2007). Therefore, *NPY5R* remains a valid candidate gene.

In general, two traditional approaches have been used to search for candidate genes for a QTL of interest (Ishikawa 2017). One approach is based on positional cloning, which aims to narrow the initial large QTL interval down to a very small interval with only one gene by the development of crossbred animals such as congenic strains or advanced intercross lines. In a previous study in mice using this approach, *Usp46* was identified as a causal gene for immobile behavior obtained by tail suspension and forced swimming tests (Tomida et al. 2009). However, positional cloning is not applicable in all cases, as it is usually difficult to narrow down to a very small interval due to low recombination in the QTL interval. The other approach is the positional and functional gene approach in which a search is made for candidate genes based on information on phenotype-related genes for which functions and expression patterns are already known. Examples of this approach have been reported for bovine *DGAT1* (Grisart et al. 2002), sheep *GDF8* (Johnson et al. 2005), and pig *IGF2* (Van Laere et al. 2003). Since this approach relies on known gene function, if the causal gene function is unknown, the true causal gene may be missed. The success stories for the two traditional approaches appear to consist of large-effect causal genes that explain 25–50% of the phenotypic variance. Compared with the two traditional approaches, our approach in the present study has the following advantages. The approach does not require narrowing down the QTL interval by further fine mapping using newly developed populations. The approach is not biased toward known gene function, in the sense that it was conducted without any prior consideration of gene function in the chick diencephalon; that is, it is a no-hypothesis approach. Furthermore, as demonstrated in the present study, the approach may be applicable to QTLs with small effects that explain less than 10% of the total phenotypic variance.

We believe that the most important aspect of our success was the use of the F_2_ segregating population for a series of analyses, which allowed us to exclude unlikely candidate genes that were included in the 95% confidence interval of the QTL at each analysis step (Fig. 1). First, the QTL location was re-analyzed using only the traits affected by the QTL, enabling us to accurately rank and select F_2_ individuals at the top and bottom extremes of the phenotypic distribution. Second, following RNA-seq analysis using two pooled RNAs of extreme individuals, quantitative real-time PCR analysis in the two parental breeds and their F_1_ individuals greatly reduced the number of candidate genes in the QTL interval from 333 to 16 genes. Third, the haplotype frequency analysis in the top and bottom F_2_ extremes allowed genes not associated with the phenotype to be filtered out, resulting in a further reduction of the number of candidate genes from 16 to 11. Finally, following an association analysis of gene expression between the two extreme groups, a conditional correlation analysis of diplotypes between gene expression and phenotype of the extreme individuals successfully revealed two strong candidate genes, *NPY5R* and *LOC101749214*. Recently, methylation QTL and expression QTL analyses were performed using the same advanced intercross population between White Leghorn and red junglefowl to map the effects of chicken domestication (Höglund et al. 2020). Thus, it was suggested that the same chicken population could be used to efficiently prioritize candidate genes. Currently, we plan to generate chickens with knockouts of the *NPY5R* and *LOC101749214* genes, respectively, using a CRISPR/Cas9 system to verify the biological functions of the two genes.

### Conclusion

By our integrated approach using an F_2_ segregating population between WL-G and NAG breed chickens, we successfully identified *NPY5R* and *LOC101749214* genes as strong candidate genes for the chromosome 4 QTL affecting innate open-field behavior, without further fine mapping the QTL interval in detail. Our integrated approach may be useful in the search for candidate genes for QTLs with small effects that explain less than 10% of the phenotypic variance in other breeds of chickens and other animal species including model animals.

## Supplementary Material

jkac316_Supplementary_Data

## Data Availability

The RNA-seq data have been deposited in the DDBJ Sequence Read Archive under the accession number DRA010275 (https://ddbj.nig.ac.jp/resource/sra-submission/DRA010275; Direct FTP site, https://ddbj.nig.ac.jp/public/ddbj_database/dra/fastq/DRA010/DRA010275). The sequence data for the SNP region of the *NPY5R* gene in WL-G and NAG breeds have been deposited in DDBJ under the accession numbers LC735722 (http://getentry.ddbj.nig.ac.jp/getentry/na/LC735722/?Format=flatfile&filetype=html&trace=true&show_suppressed=false&limit=10) and LC735723 (http://getentry.ddbj.nig.ac.jp/getentry/na/LC735723/?format=flatfile&filetype=html&trace=true&show_suppressed=false&limit=10), respectively. The phenotypic, genotypic, and other data are available at https://doi.org/10.18999/2003797. Supplemental material available at *G3* online.
