# Supplementary material for: Identification of candidate genes responsible for innate fear behavior in the chicken
Source: G3 (Bethesda). 2022 Dec 1;13(2):jkac316. doi: 10.1093/g3journal/jkac316 (PMC9911055; doi:10.1093/g3journal/jkac316)
Supplement: jkac316_Supplementary_Data [file jkac316_supplementary_data.pdf]

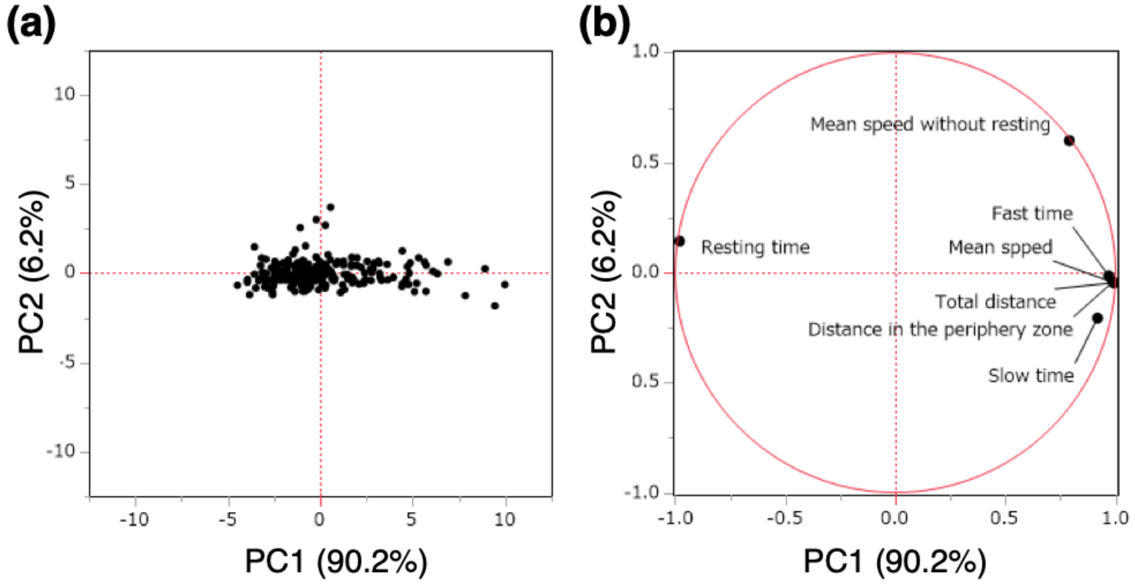

Supplementary Figure S1. (**a**) Score plot and (**b**) factor loading plot for PC1 and PC2 obtained by PC analysis using data for seven OF traits previously obtained in an F_2 population between WL-G and NAG breeds (Ishikawa *et al*. 2020). The percentage of total variance explained by each PC is shown in parenthesis.

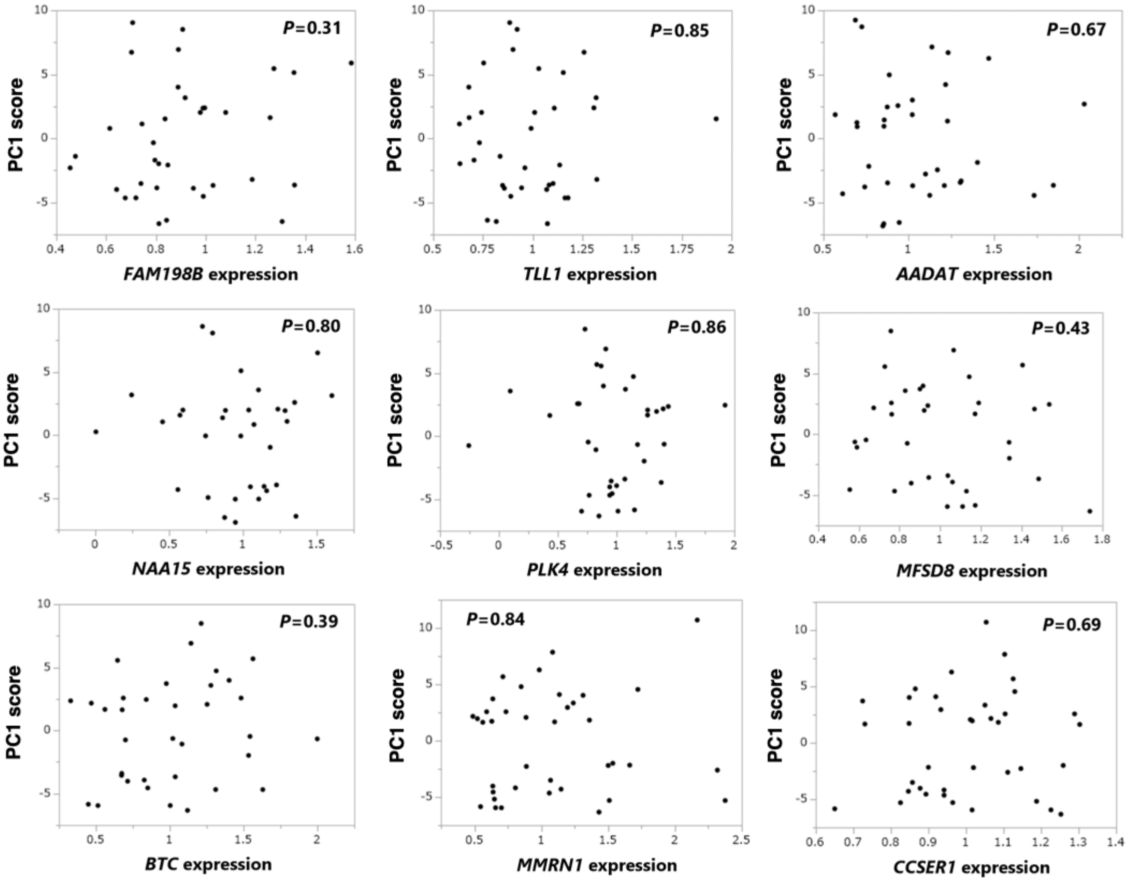

Supplementary Figure S2. A conditional correlation analysis of diplotypes between gene expression levels and PC1 scores for top and bottom F_2 individuals combined. One dot represents one individual.

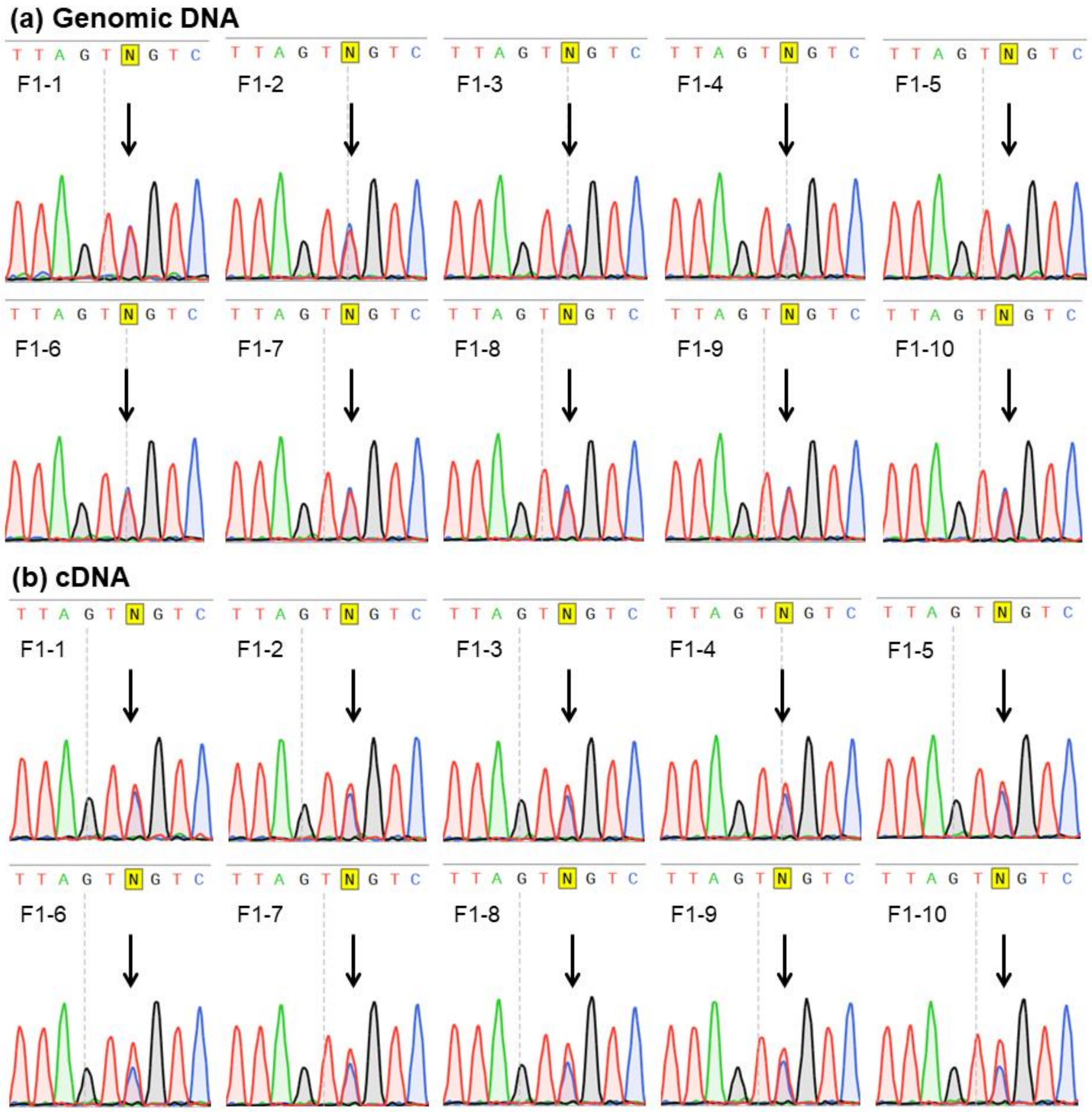

Supplementary Figure S3. Sequence results for the SNP rs316511723 region of the NPY5R gene in 10 F_1 chickens (F1-#) obtained from a cross between four WL-G females and one NAG male. (**a**) Genomic DNA. (**b**) cDNA. Arrows show the nucleotide of the SNP.

Supplementary Table S1. Primer pairs used for RT-qPCR analysis and sequence analysis.

Gene	Forward primer (5'-3')	Reverse primer (5'-3')	Reference/Accession no.	Software used
**RT-qPCR analysis**				
Pol II	AAGGAGCCGCAGGTCTAC	CTTGCTCTTTGCCGTCATAC	Ref. 1	-
TBP	TAGCCCGATGATGCCGTAT	GTTCCCTGTGTCGCTTGC	Ref. 1	-
ACTB	CCAGACATCAGGGTGTGATGG	CTCCATATCATCCCAGTTGGTGA	Ref. 2	-
GAPDH	GAAGGCTGGGGCTCATCTG	CAGTTGGTGGTGCACGATG	Ref. 2	-
ZBTB33	ACGGGCTGCAAGGTTTATG	TGCGTTTCCTGCTTGTGG	NM_001079729.1	Primer3Plus
NDUFA1	AAGGAGAAGAGGATTGCCCG	GTGGGTTCTTTGAGGTGTGC	NM_001302115.1	Primer-BLAST
NKRF	TCTGGAGCAACCACGTCTTC	CACGGAACTTAGGGACCACC	NM_001012887.2	Primer-BLAST
SLC25A43	TGGTGTAGCACAGACGCTTT	CCGAAAACAGTCAGCCATGC	XM_420343.6	Primer-BLAST
PASD1	CAAGGATGTTTACATGTGAGATTGG	GGGTGGGTGCTGGAAATAAA	XM_004940852.3	Primer3Plus
GPR50	TGGCTCCTTGCAGTATGACC	AAGTCACGATGGACAGTGGG	NM_205361.1	Primer-BLAST
MAMLD1	CCTGGACTTGTTGCCAGACT	TTCCTGCATCAGCTGTCGTT	XM_003641088.4	Primer-BLAST
LOC112532367	AAGATGCCCTCCTTCTCCTC	CACAGCGCTACACAGCATC	XR_003075046.1	Primer3Plus
TLR2A	TTCCAGTTACACAAGCCTTCCA	GCCGGTGAGCTGTATGCAT	XM_025149465.1	Primer-BLAST
SFRP2	CAATGACCTTTGCATCCCGC	TTTGTTCTTGCAGGCATCGC	NM_204773.1	Primer-BLAST
LRAT	GTCAGCTTCCAGACCGACAA	TGTTAGCCAGCCATCCACAG	XM_420371.6	Primer-BLAST
NPY2R	CCATCATCTCCTATGCCTACACC	ACACAACAACCACGCAAACC	NM_001031128.1	Primer3Plus
FAM198B	GGCTTGTTGTTTGCGTTCC	TTGTGATGTGTGTCCTCCTTTGT	XM_420360.6	Primer3Plus
NAF1	TTCATCAACTACATCGTCCTCCTC	TCATCAAGGGGCAACTCATCT	XM_015285319.2	Primer3Plus
NPY5R	ACAAAACGCAAGCAGAAGACAA	AGCGTGAAAGGAGAACAAAATAGG	XM_015285290.2	Primer3Plus
APELA	GCTCGGCAAGCAATTTCGTA	GCTCCACCAGGTCAACTGTTG	NM_001308250.1	Primer Express
TLL1	TGTGGGTGCTGTTCTTATGTAGGT	ATCTCGGTCTGGTCGTGTATGTT	NM_204703.2	Primer3Plus
PALLD	CCTGGATGGATCTAAGCCTATCA	TCAATGCCCTGTCGAAACC	XM_015285389.2	Primer-BLAST
AADAT	GAAAAGGGTTTCTTGGAGCACA	AACATGCCAGCAGCAGGAG	XM_004940922.3	Primer-BLAST
MFAP3L	AGCCCTCTCCTGAGCCTTCT	CTCCACCTGATTTTCCTGCTATG	XM_025150169.1	Primer3Plus
NAA15	ATTTCAGCAAAGGTTGCCCG	CCGGCAACTTCTTAGGGAGG	XM_015276694.2	Primer-BLAST
LOC422442	CCGGTACTTCGTCTTCAACC	AGCATAGATGCCGCTACCAG	XM_429960.6	Primer3Plus
LOC101749214	TGGAAAGCGCCAGACCA	ACCAGCAGATGTGTCGTTGTG	XM_025150043.1	Primer3Plus
SLC10A7	TTCACACCAGCGGACACAG	GATAGATGAGTAAGGGGACGGAAA	NM_001031131.1	Primer3Plus
LOC107051782	AAGCAACGAACCAGACCCAT	GGCAGCGTTTGTTCTCACAG	XR_003075024.1	Primer-BLAST
MAB21L2	ACGCTGCTGCTGTACGAGTG	CGAGGTTGGGCAGGAAGTAG	NM_204190.2	Primer3Plus
SLC20A2	TGGGATCTGTGTGGGTCTCT	AACAGCTTTCTTGGAGCGGA	NM_001305398.1	Primer-BLAST
PLK4	AAATGTTGGTTGGGCTTCGC	TGGCCATCTGGAGAGGTGTA	XM_015276633.2	Primer3Plus
MFSD8	AGGTGAACGTGCCATACTCC	GGAGTTGTTTGGGGAACGGA	XM_420463.5	Primer-BLAST
JADE1	TCAGCCCAAACAGCACTATCA	AACAACTCTCCCCTCTCACACC	XM_015276623.2	Primer3Plus
BTC	CGTCTGCGTCTGCCTCTGT	TCCGTCTCCAGCACGTCTTC	NM_001004769.1	Primer Express
MMRN1	CACCAACATTCAAAGGGATGC	TGATGAGAAGGTTTCCAGGTTGT	XM_004941034.3	Primer3Plus
CCSER1	AGCACCACCTCACTCCCTGT	GCACTGAGCATTCATCTTTTATTCC	XM_004941036.3	Primer3Plus
**Sequence analysis**				
NPY5R	TTTTTCTGCCTGGGAGGACTA	AACATCCACCGATCAAGGAG	XM_015285290.2	Primer3Plus

Ref. 1: Bélteky J, Agnvall B, Johnsson M, Wright D, Jensen P. Domestication and tameness: brain gene expression in red junglefowl selected for less fear of humans suggests effects on reproduction and immunology. R Soc Open Sci. 2016;3,(8):160033, doi:10.1098/rsos.160033

Ref. 2: Borowska D, Rothwell L, Bailey RA, Watson K, Kaiser P. Identification of stable reference genes for quantitative PCR in cells derived from chicken lymphoid organs. Vet Immunol Immunopathol. 2016;170:20-24. doi:10.1016/j.vetimm.2016.01.001

Supplementary Table S2. Effects of breed, sex and their interaction on diencephalic expression levels of 34 genes in NAG, WL-G, and their F_1 hybrids.

	**NAG**		**F_1**		**WL**		***P* value**		
**Gene**	**Male**	**Female**	**Male**	**Female**	**Male**	**Female**	**Breed**	**Sex**	**Breed × sex**
ZBTB33	1 ± 0.09	0.94 ± 0.04	0.98 ± 0.08	0.94 ± 0.05	0.99 ± 0.09	1.16 ± 0.08	0.24	0.7	0.25
NDUFA1	1 ± 0.04	1.09 ± 0.17	0.50 ± 0.04	0.47 ± 0.02	0.15 ± 0.15	0.07 ± 0.07	1.20E-08	0.95	0.71
NKRF	1 ± 0.10	1.04 ± 0.07	0.98 ± 0.09	0.88 ± 0.15	0.66 ± 0.04	0.92 ± 0.10	0.085	0.42	0.19
SLC25A43	1 ± 0.10	1.20 ± 0.06	0.66 ± 0.07	0.95 ± 0.11	0.49 ± 0.06	0.74 ± 0.06	1.60E-05	0.001	0.85
PASD1	1 ± 0.08	1.24 ± 0.09	0.98 ± 0.12	1.11 ± 0.10	0.77 ± 0.07	1.01 ± 0.08	0.057	0.013	0.8
GPR50	1 ± 0.07	1.36 ± 0.05	1.10 ± 0.08	1.23 ± 0.04	1.04 ± 0.07	1.34 ± 0.13	0.93	0.00038	0.33
MAMLD1	1 ± 0.07	1.26 ± 0.09	1.04 ± 0.09	1.15 ± 0.07	1.06 ± 0.12	1.39 ± 0.11	0.39	0.0046	0.49
LOC112532367	1 ± 0.21	1.19 ± 0.23	1.08 ± 0.25	1.00 ± 0.21	0.65 ± 0.14	1.02 ± 0.24	0.46	0.37	0.58
TMEM185A	1 ± 0.17	1.41 ± 0.09	0.87 ± 0.07	0.89 ± 0.17	0.66 ± 0.09	0.94 ± 0.04	0.0047	0.02	0.25
TLR2A	1 ± 0.04	1.03 ± 0.04	0.77 ± 0.12	0.94 ± 0.05	0.70 ± 0.10	0.86 ± 0.10	0.021	0.068	0.63
SFRP2	1 ± 0.22	1.32 ± 0.15	0.94 ± 0.18	1.09 ± 0.30	0.85 ± 0.20	0.85 ± 0.09	0.31	0.34	0.73
LRAT	1 ± 0.07	1.03 ± 0.04	1.05 ± 0.09	1.00 ± 0.07	0.89 ± 0.05	1.00 ± 0.11	0.51	0.67	0.56
NPY2R	1 ± 0.07	1.14 ± 0.09	1.12 ± 0.13	0.93 ± 0.12	0.79 ± 0.06	0.94 ± 0.04	0.084	0.65	0.13
FAM198B	1 ± 0.10	0.95 ± 0.04	0.60 ± 0.07	0.64 ± 0.10	0.44 ± 0.02	0.50 ± 0.03	5.60E-07	0.78	0.71
NAF1	1 ± 0.08	1.24 ± 0.06	1.08 ± 0.10	1.16 ± 0.11	0.93 ± 0.09	1.37 ± 0.08	0.92	0.0019	0.17
NPY5R	1 ± 0.08	0.90 ± 0.05	0.76 ± 0.05	0.76 ± 0.03	0.69 ± 0.04	0.76 ± 0.05	0.00021	0.78	0.3
APELA	1 ± 0.09	1.00 ± 0.07	1.12 ± 0.04	0.95 ± 0.10	0.91 ± 0.03	0.98 ± 0.06	0.45	0.57	0.25
TLL1	1 ± 0.03	1.12 ± 0.08	1.07 ± 0.08	1.09 ± 0.08	1.24 ± 0.03	1.22 ± 0.05	0.026	0.46	0.51
PALLD	1 ± 0.09	1.19 ± 0.11	1.00 ± 0.09	0.96 ± 0.06	0.95 ± 0.07	0.97 ± 0.06	0.19	0.37	0.35
AADAT	1 ± 0.07	1.17 ± 0.08	1.20 ± 0.08	1.45 ± 0.13	1.20 ± 0.13	1.54 ± 0.10	0.022	0.0052	0.7
MFAP3L	1 ± 0.12	1.02 ± 0.06	0.83 ± 0.05	1.00 ± 0.06	0.86 ± 0.12	1.09 ± 0.11	0.6	0.075	0.51
NAA15	1 ± 0.03	1.12 ± 0.06	0.80 ± 0.04	0.86 ± 0.04	0.92 ± 0.09	1.03 ± 0.06	0.0014	0.038	0.84
LOC422442	1 ± 0.10	1.15 ± 0.18	0.93 ± 0.09	1.09 ± 0.10	0.65 ± 0.14	1.00 ± 0.07	0.12	0.031	0.65
LOC101749214	1 ± 0.25	1.21 ± 0.10	1.01 ± 0.07	1.26 ± 0.14	1.30 ± 0.18	1.66 ± 0.14	0.047	0.045	0.89
SLC10A7	1 ± 0.08	1.18 ± 0.06	0.77 ± 0.06	1.05 ± 0.10	0.84 ± 0.09	1.16 ± 0.06	0.086	0.0004	0.65
LOC107051782	1 ± 0.18	1.34 ± 0.07	1.04 ± 0.07	1.25 ± 0.18	0.72 ± 0.05	1.15 ± 0.16	0.16	0.0047	0.69
MAB21L2	1 ± 0.14	1.10 ± 0.07	1.17 ± 0.12	1.20 ± 0.15	0.77 ± 0.12	1.05 ± 0.07	0.073	0.16	0.56
SLC20A2	1 ± 0.09	1.12 ± 0.03	1.34 ± 0.14	1.13 ± 0.19	1.00 ± 0.06	1.03 ± 0.11	0.16	0.79	0.35
PLK4	1 ± 0.08	1.11 ± 0.07	0.86 ± 0.10	0.74 ± 0.13	0.80 ± 0.09	0.87 ± 0.06	0.02	0.82	0.43
MFSD8	1 ± 0.11	1.10 ± 0.07	0.76 ± 0.09	0.86 ± 0.19	0.47 ± 0.05	0.58 ± 0.10	0.0004	0.27	1
JADE1	1 ± 0.08	1.07 ± 0.20	1.07 ± 0.11	0.98 ± 0.11	0.72 ± 0.03	0.90 ± 0.12	0.11	0.58	0.54
BTC	1 ± 0.17	1.15 ± 0.09	1.46 ± 0.15	1.74 ± 0.15	1.08 ± 0.19	1.45 ± 0.12	0.0062	0.039	0.75
MMRN1	1 ± 0.18	0.84 ± 0.15	1.86 ± 0.12	1.99 ± 0.12	2.41 ± 0.29	3.12 ± 0.22	5.20E-09	0.16	0.088
CCSER1	1 ± 0.05	0.98 ± 0.05	0.85 ± 0.05	0.85 ± 0.04	0.81 ± 0.04	0.85 ± 0.04	0.0026	0.85	0.83

Data (n=5/sex/breed) are presented as mean ± s.e.m. The *P* values were obtained by two-way ANOVA.

Supplementary Table S3. Effects of group, sex and their interactions on diencephalic expression levels of 11 genes in the top and bottom F_2 groups.

	Top		Bottom		*P* value		
**Gene**	**Male (n)**	**Female (n)**	**Male (n)**	**Female (n)**	**Group**	**Sex**	**Group × Sex**
FAM198B	1 ± 0.09 (9)	1.14 ± 0.09 (9)	0.93 ± 0.09 (10)	0.95 ± 0.10 (8)	0.19	0.41	0.54
NPY5R	1 ± 0.07 (9)	0.97 ± 0.07 (9)	0.86 ± 0.07 (10)	0.84 ± 0.07 (8)	0.067	0.73	0.92
TLL1	1 ± 0.09 (9)	0.96 ± 0.09 (9)	0.91 ± 0.08 (10)	0.99 ± 0.09 (8)	0.73	0.86	0.52
AADAT	1 ± 0.12 (9)	1.10 ± 0.12 (9)	1.07 ± 0.12 (9)	1.24 ± 0.13 (8)	0.37	0.29	0.76
NAA15	1 ± 0.25 (7)	2.65 ± 0.21 (10)	1.30 ± 0.22 (9)	2.19 ± 0.25 (7)	0.73	5.4.E-06	0.11
LOC101749214	1 ± 0.17 (8)	0.97 ± 0.16 (9)	1.34 ± 0.15 (10)	1.34 ± 0.17 (8)	0.035	0.91	0.93
PLK4	1 ± 0.38 (8)	4.21 ± 0.35 (9)	1.08 ± 0.34 (10)	3.81 ± 0.38 (8)	0.67	2.6.E-09	0.52
MFSD8	1 ± 0.10 (8)	0.84 ± 0.09 (9)	0.96 ± 0.09 (10)	0.87 ± 0.10 (8)	0.94	0.18	0.76
BTC	1 ± 0.11 (8)	0.69 ± 0.10 (9)	0.74 ± 0.10 (10)	1.04 ± 0.11 (8)	0.67	0.97	0.0077
MMRN1	1 ± 0.16 (9)	0.87 ± 0.14 (11)	1.01 ± 0.15 (10)	1.20 ± 0.17 (8)	0.28	0.85	0.31
CCSER1	1 ± 0.05 (9)	0.94 ± 0.05 (11)	0.97 ± 0.05 (10)	0.93 ± 0.06 (8)	0.74	0.36	0.82

Data are presented as mean ± s.e.m. The *P* values were obtained by two-way ANOVA.
